# Shear flow affects selective monocyte recruitment into MCP-1-loaded scaffolds

**DOI:** 10.1111/jcmm.12330

**Published:** 2014-08-08

**Authors:** Anthal I P M Smits, Virginia Ballotta, Anita Driessen-Mol, Carlijn V C Bouten, Frank P T Baaijens

**Affiliations:** a Soft Tissue Biomechanics and Tissue Engineering, Department of Biomedical Engineering, Eindhoven University of TechnologyEindhoven, The Netherlands; b Institute for Complex Molecular Systems (ICMS), Department of Biomedical Engineering, Eindhoven University of TechnologyEindhoven, The Netherlands

**Keywords:** *in situ* tissue engineering, cardiovascular, chemotaxis, CCL2, bioactive scaffold, immunomodulation, peripheral blood mononuclear cells, angiogenic monocytes, haemodynamics

## Abstract

Novel cardiovascular replacements are being developed by using degradable synthetic scaffolds, which function as a temporary guide to induce neotissue formation directly *in situ*. Priming of such scaffolds with fast-releasing monocyte chemoattractant protein-1 (MCP-1) was shown to improve the formation of functional neoarteries in rats. However, the underlying mechanism has not been clarified. Therefore, the goal of this study was to investigate the effect of a burst-release of MCP-1 from a synthetic scaffold on the local recruitment of circulating leucocytes under haemodynamic conditions. Herein, we hypothesized that MCP-1 initiates a desired healing cascade by recruiting favourable monocyte subpopulations into the implanted scaffold. Electrospun poly(ε-caprolactone) scaffolds were loaded with fibrin gel containing various doses of MCP-1 and exposed to a suspension of human peripheral blood mononuclear cells in static or dynamic conditions. In standard migration assay, a dose-dependent migration of specific CD14^+^ monocyte subsets was observed, as measured by flow cytometry. In conditions of pulsatile flow, on the other hand, a marked increase in immediate monocyte recruitment was observed, but without evident selectivity in monocyte subsets. This suggests that the selectivity was dependent on the release kinetics of the MCP-1, as it was overruled by the effect of shear stress after the initial burst-release. Furthermore, these findings demonstrate that local recruitment of specific MCP-1-responsive monocytes is not the fundamental principle behind the improved neotissue formation observed in long-term *in vivo* studies, and mobilization of MCP-1-responsive cells from the bone marrow into the bloodstream is suggested to play a predominant role *in vivo*.

## Introduction

Recognition of the role of monocytes in natural wound healing has been the basis of novel regenerative therapies, such as *in situ* cardiovascular tissue engineering. This approach aims at the regeneration of living cardiovascular substitutes directly within the body. Starting from the implantation of bare biodegradable constructs, these therapies rely on *in situ* colonization by host cells and subsequent neotissue formation as the original construct is degraded 1,2.

Monocytes are circulating leucocytes that actively contribute to homoeostasis and innate immune processes *via* phagocytosis and cytokine release 3. Originating from bone marrow precursor cells or from splenic reservoirs, they respond to inflammatory signals, migrating from the bloodstream into peripheral tissues, in which they differentiate into macrophages or dendritic cells 4. The diversity of physiological roles played by monocytes is reflected in their morphological heterogeneity 5. Three distinct populations were identified in humans, based on their expression of the surface markers CD14, CD16 and CCR2 6–8. Classical CD14^+^/CD16^−^/CCR2^+^ monocytes represent the most abundant subset, consisting of highly versatile cells involved in phagocytic and pro-inflammatory activities, as well as in tissue repair and angiogenesis 9. The non-classical CD14^dim^/CD16^+^/CCR2^−^ population comprises monocytes with a patrolling function, exhibiting high motility in the local surveillance of tissues, which is attributed to an enriched expression of genes involving cytoskeletal rearrangement 9,10. Intermediate CD14^+^/CD16^+^/CCR2^+^ monocytes display enhanced expression of angiogenic and anti-inflammatory markers, which suggests their reparative potential 6,11. Monocyte mobilization and trafficking during homoeostasis and inflammation occurs in response to local stimuli in conformity with the leucocyte adhesion cascade 12. Chemokines, such as fractalkine (CX_3_CL1) and stromal cell-derived factor-1α (SDF-1α or CXCL12), were shown to mediate migration and arrest of the non-classical and intermediate monocytes, which highly express the respective receptors CX_3_CR1 and CXCR4 13. In contrast, monocyte chemoattractant protein-1 (MCP-1 or CCL2), mediates recruitment of CCR2^+^ monocytes (*i.e*. classical and intermediate monocytes), as well as related circulating progenitor species, such as CD34^+^/CD45^+^ fibrocytes, which are known to promote repair and remodelling 14–16. In addition, chemokine signalling can enhance monocyte anchorage to the endothelium, by inducing morphological changes in their α_4_- and β_2_-integrins, a pathway that is further influenced by physiological shear stresses exerted by the flowing blood 17,18.

Recent progress in the field of *in situ* tissue engineering was achieved by Shin’oka *et al*., who successfully treated congenital cardiac defects in humans with synthetic vascular grafts, preseeded on-the-fly with autologous bone marrow cells in a single operation 19,20. The process that drove the transformation of the graft into a functional neovessel was explained *via* animal studies as a positive inflammatory response, induced by the scaffold and stirred by infiltration of host monocytes. This process was enhanced by paracrine factors secreted by the preseeded cells, of which MCP-1 was identified as one of the principal mediators 21. Additionally, it was suggested that a burst-release of MCP-1 from functionalized, acellular constructs prompted the enhanced recruitment of immune cells in the early-phase, leading to long-term vascular remodelling 21. However, a mechanistic understanding of the cellular events behind this process is lacking, which is fundamental for further clinical translation.

Our goal here was to elucidate the initial response of circulating immune cells to a MCP-1-loaded scaffold under physiological conditions of flow. We hypothesized that MCP-1 induces a favourable healing cascade at time of implantation by selectively attracting angiogenic and reparative circulating species, such as CCR2^+^ monocytes and fibrocytes. To test this, we developed a hybrid scaffold consisting of a highly porous electrospun poly(ε-caprolactone; PCL) structure, combined with a fibrin gel containing rapidly releasing MCP-1. We first established the chemotactic effect of our bioactive scaffolds, loaded with varying doses of MCP-1, on specific mononuclear cell populations using static chemotaxis assays. These scaffolds were subsequently placed in a previously validated *in vitro* flow setup to investigate its selectivity in recruiting cells in physiologically relevant conditions of pulsatile flow 22.

## Materials and methods

### Electrospinning

Fibrous PCL scaffolds were prepared by electrospinning by using a climate-controlled electrospinning apparatus (EC-CLI; IME Technologies, Geldrop, the Netherlands). A viscous polymer solution was prepared by dissolving PCL (Purasorb, Purac Biomaterials (Gorinchem, the Netherlands), density ρ_PCL_ = 1.15 kg/m^3^) in chloroform (20% w/w). The solution was fed through a laterally translating nozzle (18 gauge), to which a high voltage of 16 kV was applied, at a flow rate of 25 μl/min. Polymer fibres were collected on a grounded rotating cylindrical drum (Ø32 mm) at 13 cm distance. Temperature and relative humidity were controlled at 23°C and 50% respectively. Overall scaffold thickness was controlled *via* the total spinning time. The resulting PCL sheets were placed under vacuum overnight to remove any remaining solvent. The average scaffold thickness was measured per electrospun sheet (~8–12 measurements at arbitrary locations per sheet; 10 sheets in total) by using a digital microscope (VHX-500FE; Keyence, Osaka, Japan). The overall density of the electrospun scaffolds ρ_0_ was determined gravimetrically measuring the weight and the thickness of mesh samples over a defined area. The scaffold porosity was calculated by using equation (1):

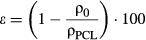
(1)

The fibre diameter and inter-fibre distance were measured by using scanning electron microscopy (SEM) in high vacuum with an electron beam of 1.0–2.0 kV (Quanta 600F; FEI, Hillsboro, OR, USA). Per sheet, three SEM images were taken at random locations (magnification 500×) and the respective averages were calculated from 25 to 30 measurements per image.

### MCP-1 loading

Scaffolds were sterilized by using 70% ethanol, followed by washing in PBS, and overnight incubation in complete medium, consisting of RPMI (RPMI 1640; Gibco, Carlsbad, CA, USA), supplemented with 10% foetal bovine serum (FBS Gold; PAA, Cölbe, Germany) and 1% penicillin/streptomycin (Lonza, Basel, Switzerland), to allow for adsorption of serum proteins and increase the hydrophilicity of the scaffold. Recombinant human MCP-1 (Peprotech, Rocky Hill, NJ, USA) was mixed into a sterile fibrinogen solution (10 mg fibrinogen/ml) at final concentrations of 2, 20 or 50 ng/ml. The fibrinogen, containing MCP-1 or PBS for controls, was mixed with thrombin (10 IU/ml) and immediately seeded into the scaffolds.

### MCP-1 release

To measure the release rate of MCP-1, scaffolds were loaded with fibrin and MCP-1 to a final concentration of 20 ng/ml of medium and exposed to RPMI medium in both static and flow conditions. The applied flow conditions were similar to the conditions used in the cell experiments with a pulsatile flow of 1 Hz, a peak pressure of 100 mmHg and peak shear stress of 1.6 Pa on the scaffold surface. Medium samples were collected at time-points 20 min., 45 min., 90 min., 3 hrs, 6 hrs and 24 hrs. MCP-1 content was measured by using an ELISA kit (RayBiotech, Norcross, GA, USA), according to the manufacturer’s protocol. The resulting release rate was determined cumulatively per scaffold (*N* = 6 per group).

### Cell isolation

Human peripheral blood buffy coats were obtained from 11 healthy donors (ages 21–60 years) under informed consent (Sanquin, Nijmegen, the Netherlands). Buffy coats were diluted in 0.6% sodium citrate in PBS, after which the mononuclear cell fraction (human peripheral blood mononuclear cells, hPBMC) was isolated by using density gradient centrifugation on iso-osmotic medium with a density of 1.077 g/ml (Lymphoprep, Axis-Shield, Oslo, Norway). After washing, the hPBMC were resuspended in freezing medium consisting of RPMI, supplemented with 20% FBS and 10% Dimethyl sulfoxyde (Merck Millipore, Amsterdam, the Netherlands), and cryopreserved in the vapour phase of liquid nitrogen. Before use, cells were rapidly thawed, counted and resuspended in complete RPMI medium at a concentration of 5 × 10^6^ cells/ml. For each donor, hPBMC were characterized with flow cytometry and qPCR as described below.

### Cell characterization

Cells were double-stained for CD14/CD16, CD4/CD8 and CD34/CD45, by using the following conjugated monoclonal antibodies: anti-CD14 (FITC; AbD Serotec, Raleigh, NC, USA); anti-CD16 (RPE-Cy5 or Alexa 647; AbD Serotec); anti-CD4 (FITC; Diaclone, Besancon Cedex, France); anti-CD8 (PE; Diaclone); anti-CD34 (PerCP-Cy5.5; BD Biosciences, San Jose, CA, USA); anti-CD45 (FITC; BD Biosciences). Cell viability was assessed by using 7-amino-actinomycin-D (7AAD; eBioscience, San Diego, CA, USA). Unspecific binding was blocked by using 0.5% bovine serum albumin (Sigma-Aldrich, St. Louis, MO, USA) in PBS. To increase the specificity for rare progenitor cell detection, an additional blocking step was performed with human FcR Blocking Reagent (Miltenyi Biotech, Leiden, the Netherlands). After washing, labelled cells were measured by using a bench-top flow cytometer (Guava easyCyte 6HT; Merck Millipore) until 20,000 events per sample. Data analysis was performed by using the Guava Express Pro software package, combined with FCS Express (De Novo Software, Los Angeles, CA, USA). To accommodate for inter-donor variations within the relative fractions of the various cell populations, absolute cell numbers were normalized per donor on the specific baseline value of each cell type.

### Chemotaxis assays

Cell migration was assessed by using Boyden chambers in a 24-wells plate fitted with a transparent PET membrane with 3 μm pores (ThinCerts; Greiner Bio-One, Frickenhausen, Germany). Seeded scaffolds were placed in the bottom compartment, with a MCP-1 concentration of 0, 2, 20, or 50 ng/ml, based on previous reports 21,23,24 (*N* = 8 per group). Unseeded electrospun PCL and fibrin only were included as controls (*N* = 4 per group). Scaffolds were covered in 800 μl of complete medium. The chemotactic effects of the scaffolds were compared with the effects of MCP-1 directly dissolved in complete medium, at the same concentrations, but without scaffolds (*N* = 8 per group). hPBMC were added to the top compartment at a concentration of 5 × 10^6^ hPBMC/ml in a total volume of 500 μl. After 4 hrs of incubation (37°C; 5% CO_2_), the migrated cells in the bottom compartment were analysed with flow cytometry. The scaffolds were stained with 10 μM CellTracker Green (CTG; 5-Chloromethylfluorescein Diacetate; Molecular Probes, Eugene, OR, USA) for immediate visualization of adherent cells. Three independent experiments were conducted, each by using hPBMC from a different donor.

### Flow experiments

Cell recruitment under conditions of pulsatile flow was studied by using a previously developed mesofluidics setup 22. In brief, scaffold strips seeded with 200 ng MCP-1 were placed in a custom parallel-plate flow chamber, in which they were exposed to a recirculating suspension of hPBMC in 10 ml of complete RPMI medium (5 × 10^6^ hPBMC/ml). The total concentration of MCP-1 per flow chamber was therefore 20 ng/ml. Scaffolds seeded with fibrin only served as controls. A pulsatile flow of 1 Hz was imposed, with a peak pressure of 100 mmHg and peak shear stress of 1.6 Pa on the scaffold surface, mimicking average physiological conditions for small-diameter arteries. Four independent experiments were conducted with hPBMC from seven different donors (*N* = 16 per group). Samples of the circulating cell suspension were taken aseptically, at time-points 0, 1, 2.5, 6, 8 and 24 hrs, without stopping the flow, *via* in-line Luer injection ports (ibidi GmbH, Martinsried, Germany). To focus on early effects, two independent experiments with hPBMC from two different donors (*N* = 5 per group) were run for 4 hrs and samples were taken at time-points 0, 10, 30, 60 and 240 min. After 4 or 24 hrs, the scaffolds were sacrificed for analysis and fixated overnight in 3.7% formaldehyde (Merck Millipore) for immunostainings or 2.5% glutaraldehyde (Grade I; Sigma-Aldrich) for SEM analysis, or snap-frozen in liquid nitrogen and stored at −80°C for qPCR analysis. To serve as gene expression controls, hPBMC from the initial suspension of each donor were stored at −80°C in lysis buffer (Buffer RLT; Qiagen, Venlo, the Netherlands). To accommodate for inter-donor variations in the gene expression levels, hPBMC from an external donor were stimulated with 1 μg/ml lipopolysaccharide (LPS, *Escherichia coli*; Sigma-Aldrich) for 30 min. to activate the cells to express inflammatory genes, which were used as reference values for normalization.

### Immunostainings

Formaldehyde-fixated samples were washed in PBS and permeabilized in 0.5% Triton X-100 in PBS (Merck Serono). Non-specific binding was blocked by incubation in 10% horse serum (Invitrogen, Bleiswijk, the Netherlands) in PBS. Cells were incubated overnight at 4°C with primary antibodies against CD68 (1:100; AbD Serotech), CCR7 (1:100; Abcam, Cambridge, UK) and CD163 (1:100; AbD Serotech) in 1% bovine serum albumin in PBS. The scaffolds were then washed and incubated for 60 min. with Alexa fluor 555 (1:300) for CD68, Alexa fluor 647 (1:300) for CCR7 and Alexa fluor 488 (1:300) for CD163. Scaffolds were subsequently stained with 4′,6-diamidino-2-phenylindole (DAPI; Sigma-Aldrich). After washing steps, scaffolds were mounted on slides with Mowiol (Calbiochem, San Diego, CA, USA) and observed with a confocal microscope (TCS SP5X; Leica Microsystems, Wetzlar, Germany).

### SEM

Glutaraldehyde-fixated samples were washed in PBS and dehydrated in a graded ethanol series, starting from 50% to 100% ethanol in 5–10% increments. Samples were visualized in high vacuum with an electron beam of 1.0–2.0 kV (Quanta 600F; FEI).

### qPCR and gene expression analysis

Scaffolds were disrupted with a microdismembrator (Sartorius, Goettingen, Germany) and RNA was subsequently isolated with Qiagen RNeasy kit (Qiagen) according to the manufacturer’s instructions. cDNA was synthesized with 50 ng RNA by using M-MLV Reverse Transcriptase (Invitrogen). Expression levels of genes involved in the inflammatory process were evaluated with SYBR®Green Supermix (Bio-Rad, Hercules, CA, USA) with CFX384 real-time detection system (Bio-Rad, Hercules, CA, USA), and GAPDH was selected as reference gene. Primer sequences of selected genes are provided in Table S1 of supplemental data. C_t_ values were normalized to the reference gene and to the LPS-treated hPBMC to obtain the relative gene expression. The expression levels of LPS-treated cells were set to a value of 1 for all genes, to serve as reference for the activated hPBMC state 25.

### Statistical analysis

Data collected with flow cytometry and qPCR are expressed as mean ± standard error of the mean. When variances could not be considered equal (for the flow cytometry data), a logarithmic transformation was applied. An anova with Bonferroni post-hoc testing was performed to detect statistical differences between the groups. Because of a non-normal distribution of the data from the gene expression analyses, these data were analysed with Kruskal–Wallis tests followed by Dunn’s multiple comparison tests. Statistical analyses were performed with Prism software (GraphPad, La Jolla, CA, USA) and differences were considered significant for *P*-values <0.05.

## Results

### Hybrid PCL/fibrin scaffolds demonstrate a burst-release of MCP-1

**Figure 1:**
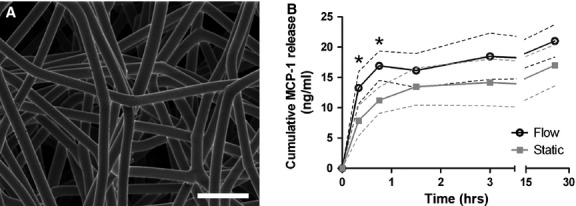
Scaffold characterization and MCP-1 release profile. (**A**) SEM image of an electrospun polycaprolactone scaffold with an average fibre diameter of 10.9 ± 0.8 μm. Scale bar represents 50 μm. (**B**) Cumulative release of MCP-1 from the PCL/fibrin construct measured over 24 hrs in static and pulsatile flow conditions. The results demonstrate a burst-release of MCP-1 in the first 45 and 90 min. for flow and static conditions, respectively. The data are plotted as mean ± SD; *P < 0.05.

Electrospinning resulted in isotropic fibrous PCL scaffolds with a fibre diameter distribution of 10.9 ± 0.8 μm and an inter-fibre distance of 119 ± 39 μm (Fig. 1A). For the chemotaxis experiments, the average scaffold thickness was 392 ± 36 μm, to prevent direct contact with the membrane. For the flow experiments, scaffold thickness was 507 ± 79 μm. The overall scaffold density ρ_0_ was determined to be 0.12 kg/m^3^, from which the scaffold porosity was calculated to be ~90% [Eq. (1)].

In static conditions, seeding the scaffolds with MCP-1 in fibrin gel resulted in a burst-release of MCP-1 in the first 90 min., followed by a gradual protein release up to 24 hrs. In flow conditions, the protein release was significantly accelerated up to 45 min., after which the release rate showed a gradual increase similar to the static release (Fig. 1B).

### Baseline cell composition comprises biological inter-donor variations

**Figure 2:**
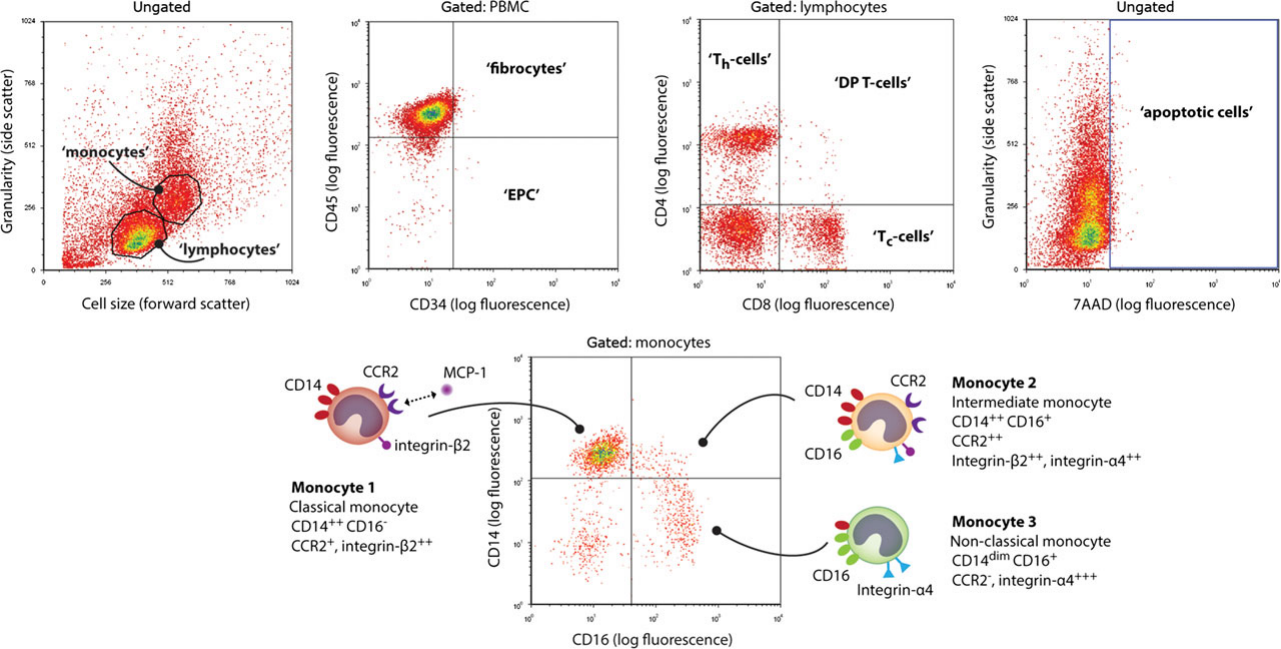
Gating strategy adopted to characterize hPBMC populations. (**A**) Cell populations were quantified by gating the lymphocyte and monocyte clouds in the forward scatter-side scatter plots, which combined formed the total hPBMC gate. (**B**) Within this gate, CD34^+^/CD45^−^ and CD34^+^/CD45^+^ cells were classified as ‘endothelial progenitor cells’ (EPC) and ‘fibrocytes’ respectively 15,26,27. (**C**) Lymphocytes were further characterized for the presence of helper T cells (Th cells; CD4^+^/CD8^−^), cytolytic T cells (T_c_-cells; CD4^−^/CD8^+^), and double-positive T cells (DP T cells; CD4^+^/CD8^+^) 28. (**D**) Overall cell viability was quantified *via* 7AAD labelling. (**E**) Characterization of monocytes subsets based on CD14 and CD16 labelling, with cartoons illustrating the specific surface receptors and integrins. Monocytes subsets were specified as monocyte 1 (Mon1; CD14^+^/CD16^−^), monocyte 2 (Mon2; CD14^+^/CD16^+^), monocyte 3 (Mon3; CD14^dim^/CD16^+^) 6.

Baseline values for the initial hPBMC population were determined for each donor by using flow cytometry, as specified in Figure 2 6,15,26–28. The cellular composition displayed biological inter-donor variations, and baseline values of all specified cell types fell within physiological values for healthy adults, as reported elsewhere (Table 1) 6,27–29.

**Table 1:** Baseline cell composition of the hPBMC

	Mean ± SD	Range (min–max)
PBMC (μl^−1^)	2666 ± 927	1443–4736
Monocytes (% of PBMC)	21.9 ± 10.5	7.9–37.5
Mon1 (% of monocytes)	81.0 ± 8.4	67.2–94.2
Mon2 (% of monocytes)	5.2 ± 3.0	1.2–10.2
Mon3 (% of monocytes)	13.8 ± 5.8	4.6–22.6
Lymphocytes (% of PBMC)	78.1 ± 10.5	62.5–92.1
T_h_ cells (% of lymphocytes)	39.2 ± 13.6	18.0–58.5
T_c_ cells (% of lymphocytes)	27.1 ± 8.2	17.1–39.1
DP T cells (% of lymphocytes)	1.2 ± 0.7	0.6–2.5
CD4/CD8 ratio (-)	1.6 ± 0.8	0.5–2.8
Fibrocytes (% of PBMC)	1.7 ± 0.4	1.0–2.4
EPC (% of PBMC)	0.13 ± 0.05	0.05–0.22

Mean values of initial cell populations among 11 donors obtained *via* flow cytometry. All values fell within physiological ranges.

### MCP-1-loaded scaffolds induce highly specific chemotaxis of monocyte subsets

**Figure 3:**
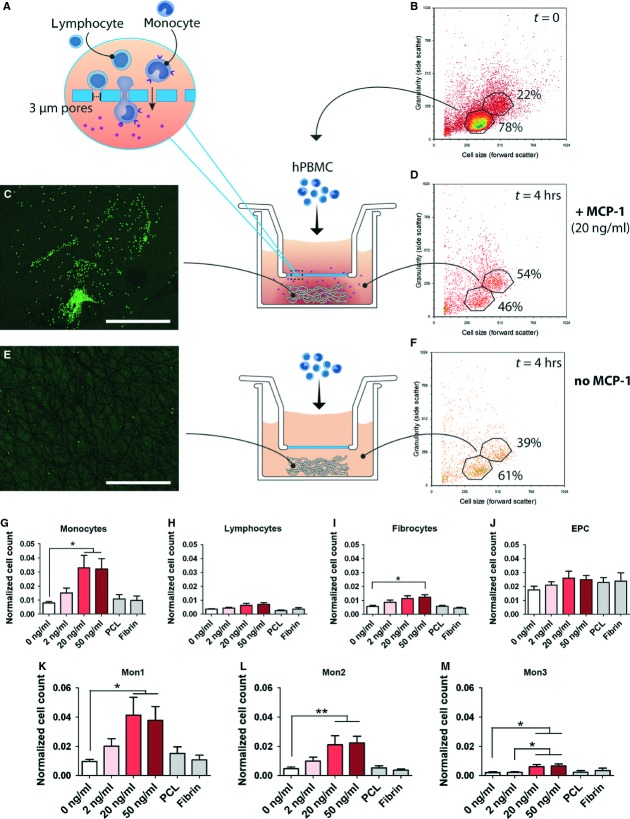
Chemotaxis assays revealed enhanced migration of Mon1 and Mon2 subsets towards MCP-1 concentrations of 20 and 50 ng/ml. (**A**) Schematic of monocyte migration through 3 μm pores of a Thincert™ membrane. (**B**) Flow cytometry showing the initial hPBMC composition, seeded into the upper compartment. (**C** and **D**) Enhanced migration of monocytes towards MCP-1-loaded scaffolds after 4 hrs as revealed by immunofluorescence of the construct (**C**) and by flow cytometry of migrated cells in the bottom compartment (**D**). (**E** and **F**) CTG-staining showing a limited adhesion of cells onto the scaffolds without MCP-1 (**E**), and relative flow cytometry revealing limited increase of monocyte fraction in the migrated population (**F**). (**G**–**M**) Quantification of migrated populations in response to various doses of MCP-1 incorporated into PCL/fibrin scaffolds. Cell counts were normalized per cell type on the initial cell count of that specific population. Scale bars represent 500 μm. *P < 0.05; **P < 0.01.

After 4 hrs, the migrated cell population contained an increased fraction of monocytes compared with the initial hPBMC suspension in all groups (Fig. 3B, D, F). CTG-staining demonstrates increased adhesion of migrated cells onto the scaffolds loaded with MCP-1, as compared with scaffolds containing only fibrin (Fig. 3C and E). Incorporation of MCP-1 into the PCL/fibrin scaffolds led to a significant increase in migrated monocytes for concentrations of 20 and 50 ng/ml MCP-1, while overall lymphocyte migration was less than 1% of the initial lymphocytes for all conditions (Fig. 3G and H). No significant effect of MCP-1 loading was observed on the migration of fibrocytes and EPC (Fig. 3I and J). Monocyte subsets mon1 and mon2 were highly responsive to MCP-1 at concentrations of 20 and 50 ng/ml, while overall migration of mon3 was very limited for all conditions tested (Fig. 3K–M). In comparison with the fibrin-seeded control scaffolds, mon2 showed the most enhanced migration with over sixfold increase in mon2 cell counts over the 0 ng/ml scaffolds, compared with a nearly fourfold increase in mon1 for MCP-1 concentrations of 20 and 50 ng/ml. The presence of PCL or fibrin alone did not lead to any changes in cell migration compared with the controls with 0 ng/ml MCP-1 (Fig. 3G–M). Overall, the migration profile towards the MCP-1-loaded scaffolds was similar to results obtained by direct addition of MCP-1 to culture medium (Fig. S1).

### Flow experiments

#### MCP-1 release does not result in selective recruitment under conditions of flow

**Figure 4:**
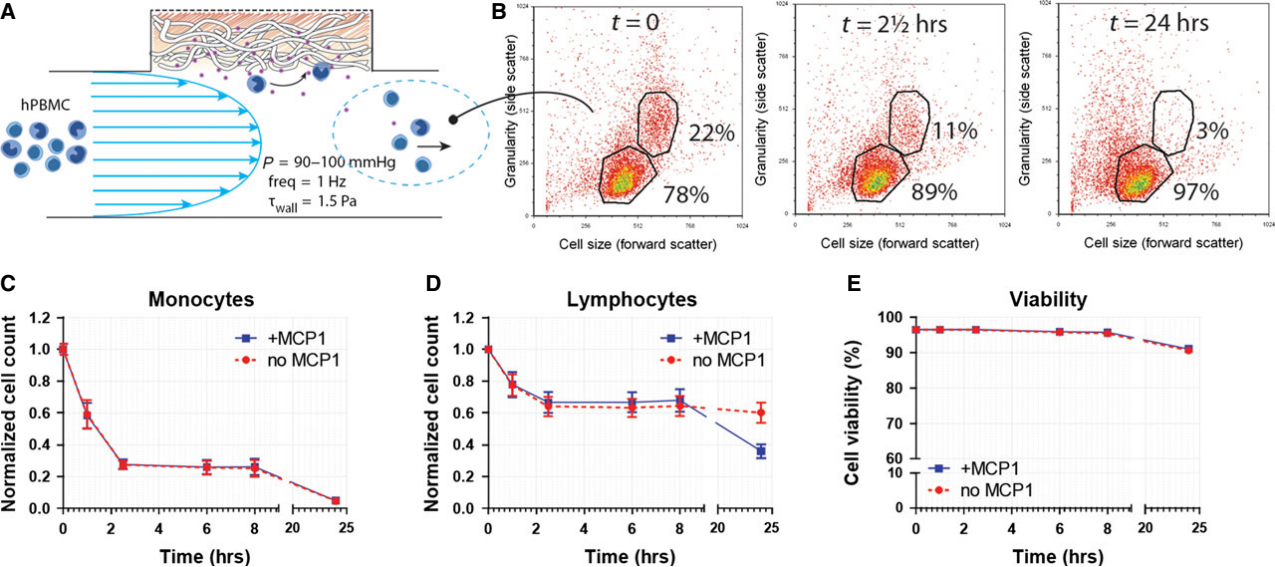
Cell recruitment by MCP-1 in scaffolds under physiological flow did not result in selective infiltration of monocytic subsets in response to MCP-1 at time-points beyond 1 hr. (**A**) Drawing representing circulating hPBMC exposed to PCL/fibrin scaffolds with and without MCP-1 in conditions of physiological flow. (**B**) Flow cytometry of the circulating hPBMC suspension, showing monocyte depletion over time. (**C** and **D**) Analysis of circulating cell populations *via* flow cytometry revealing no differences between MCP-1 loaded scaffold and controls. Cell counts were normalized per cell type on the initial cell count of that specific population. (**E**) Viability of circulating cells was not affected by flow within the 24 hr follow-up.

**Figure 5:**
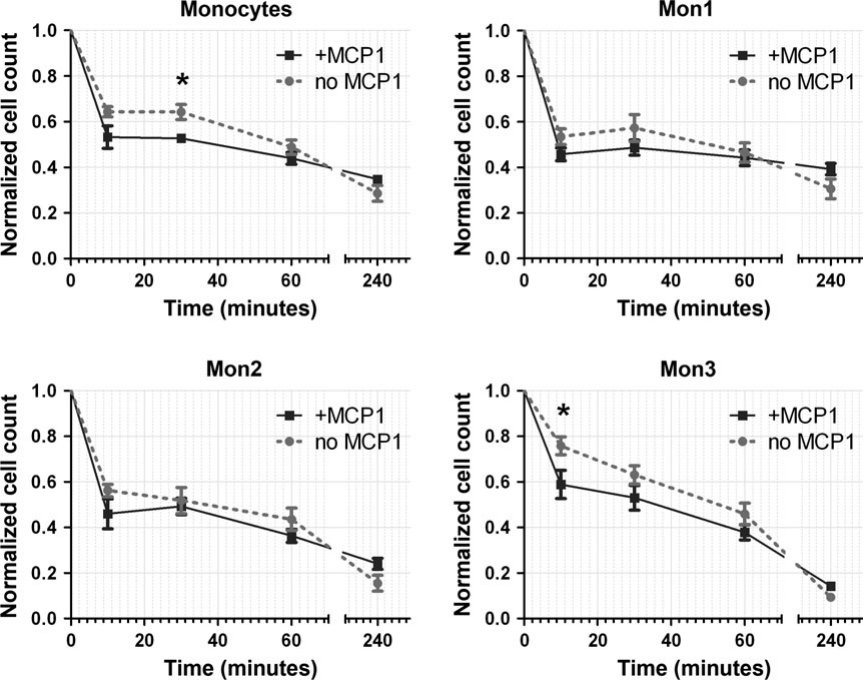
Accelerated monocyte recruitment by MCP-1-loaded scaffolds within the first 30 min. of flow. (**A**–**D**) Flow cytometric analysis of monocyte subsets revealed a significantly enhanced depletion of total monocyte counts after 30 min. of flow in response to MCP-1. All differences between the MCP-1-loaded scaffolds compared with the control scaffolds were negated beyond the 1 hr time-point. *P < 0.05.

Based on the results obtained from the chemotaxis assays, a MCP-1 concentration of 20 ng/ml was selected for loading the scaffolds in the flow experiments. Flow cytometry of the remaining cell suspension revealed that after 24 hrs, the circulating hPBMC consisted almost exclusively of lymphocytes (Fig. 4A and B). The analysis per cell type indicated that lymphocyte count remained relatively stable at ~65% of the original amount, while monocytes were rapidly depleted to 27% of the initial monocyte count within the first 2.5 hrs of flow, and to 5% after 24 hrs (Fig. 4C and D). Fibrocytes and EPC demonstrated an immediate depletion within the first hour, to ~45% and 35% of their initial cell numbers, respectively, after which their numbers gradually decreased (data not shown). Viability was not affected under any of the experimental conditions, with an overall viability of >90% throughout the course of the experiment (Fig. 4E). Surprisingly, within this timeframe, no significant differences were observed between the MCP-1 and control groups for any of the cell types studied. Therefore, a second series of experiments was conducted focusing on the immediate events in the first hour. These short-term follow-up experiments revealed an accelerated depletion of all monocytes after 10 and 30 min. of flow in response to MCP-1 (Fig. 5A–D). After 4 hrs of flow, the remaining relative fractions of monocyte subsets mon2 and mon3 were lower compared with mon1, indicating a more pronounced depletion of mon2 and mon3 within this timeframe.

#### Scaffolds induce monocyte-to-macrophage differentiation with mixed phenotype

**Figure 6:**
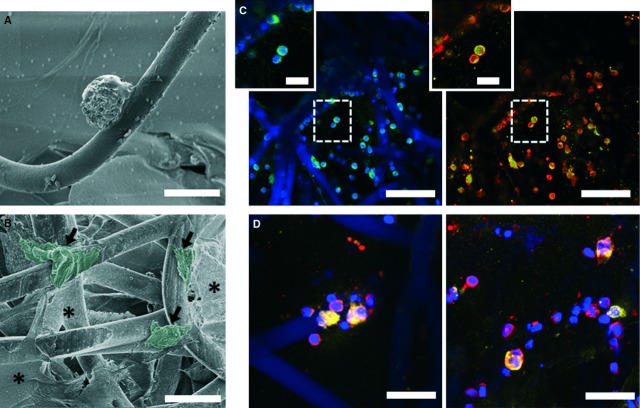
Monocytes were recruited to scaffold/fibrin constructs and resulted in M1 and M2 activated macrophages. Monocyte adherent on a PCL fibre (**A**) and activation of monocytes into macrophages (**B**) visualized with SEM. (**C** and **D**) Immunofluorescence of CD68^+^ macrophages (green), with expression of inflammatory (CCR7^+^; red) and wound healing features (CD163^+^, yellow). Cell nuclei are shown in blue (DAPI). (**A** and **B**) Scale bars represent 25 μm; arrows indicate macrophages, artificial colour overlay was added by using Adobe Photoshop in post-processing to highlight macrophage morphology; * indicates fibrin. (**C** and **D**) Scale bars represent 50 μm (**C**), 10 μm (insets) and 25 μm (**D**).

Analysis of the scaffolds after 24 hrs of flow exposure revealed monocyte activation and adhesion to the scaffold (Fig. 6A). Furthermore, macrophage differentiation was observed, represented by large, irregular-shaped cells, spreading along the PCL fibres, as visualized by SEM (Fig. 6B). Consistently, immunofluorescent analysis demonstrated abundant infiltration of CD68^+^ macrophage into the scaffolds. Small, rounded, CD68^−^/CCR7^+^ lymphocytes were sparsely detected. Macrophages displayed a predominant M1 phenotype with strong, but not exclusive, expression of CCR7. In addition, macrophage polarization towards the M2 phenotype was detected, characterized by CD163 expression (Fig. 6C). This marker was mainly observed in morphologically larger or fused cells (Fig. 6D). No apparent difference was observed between the MCP-1 and control groups.

#### Cell recruitment under flow induces up-regulation of immunomodulatory genes

**Figure 7:**
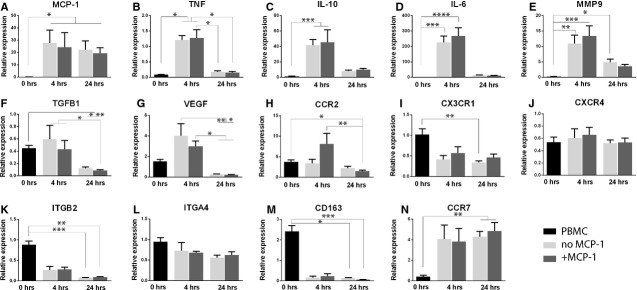
Gene expression of immunomodulating and angiogenic markers was up-regulated in recruited hPBMC after 4 hrs of flow. (**A–E**) Compared with LPS-stimulated cells, the initial hPBMC population showed low expression of genes involved in immunomodulation, such as MCP-1, TNF, IL10, IL6 and MMP9. Up-regulation of these genes was observed at the 4 hrs time-point, followed by a decrease at 24 hrs. MCP-1 was significantly up-regulated for recruited cells at all time-points. (**F**) TGFB1 expression was down-regulated compared with the LPS control and no substantial changes were observed between the initial population and the cells recruited after 4 hrs, while expression decreased significantly at 24 hrs. (**G**) The angiogenic marker VEGF showed up-regulation after 4 hrs, followed by a significant decrease at 24 hrs compared with 4 hrs. (**H–J**) Gene expression of CCR2, CX_3_CR1 and CXCR4 was evaluated, representing the receptors for the signalling molecules MCP-1, fractalkine and SDF-1α, respectively. CCR2 was up-regulated in all groups, as compared with the LPS control, and exhibited increased expression in the MCP-1 group at 4 hrs. CX_3_CR1 was down-regulated for recruited cells at both time-points, while CXCR4 expression showed a decrease with respect to the LPS-treated cells, but no significant differences between the groups. (**K** and **L**) Concerning expression of integrins, no differences were detected between the controls and the initial hPBMC population, while recruited cells displayed a strong down-regulation of ITGB2 at 4 and 24 hrs. No differences were notable for ITGA4 in any group. (**M**) Expression of anti-inflammatory macrophage marker CD163 was high for cells at the initial time-point compared with the LPS-treated reference cells, but its expression decreased significantly for infiltrating hPBMC. (**N**) Opposite behaviour was observed for the pro-inflammatory marker CCR7, which showed a low expression by the initial cell population compared with the LPS-treated hPBMC, followed by a large increase in expression for recruited cells at both time-points. *P < 0.05; **P < 0.01; ***P < 0.001; ****P < 0.0001.

Gene expression of recruited cells in the scaffold after 4 and 24 hrs of flow is depicted in Figure 7. Overall, MCP-1 loading did not have a significant effect on gene expression, with exception of CCR2 expression, which was significantly up-regulated at 4 hrs in the MCP-1-loaded scaffolds. Shear flow, on the other hand, generally resulted in an up-regulation of immunomodulatory and angiogenic genes, compared with LPS-activated hPBMC, with increased expression at 4 hrs compared with 24 hrs. Furthermore, flow led to a decrease in integrin expression of ITGB2 over time, but not of ITGA4. Expression of the anti-inflammatory macrophage marker CD163 was significantly down-regulated in response to flow, while the pro-inflammatory macrophage marker CCR7 was significantly up-regulated. Expression of IL-4, IL-13, MRC-1, and CXCL12 was undetectable (data not shown).

## Discussion

Recent studies aimed at the *in situ* regeneration of cardiovascular tissues have demonstrated *de novo* tissue formation on a degradable synthetic starter matrix, driven by the host immune response 19–21,30,31. It was postulated that MCP-1 is one of the key mediators in the process and, as such, may be used as a therapeutic agent 21. Concurrently, we recently demonstrated improved neotissue formation in MCP-1-loaded PCL/fibrin scaffolds in rats, but the underlying mechanism has not yet been clarified (H. Talacua, A.I.P.M. Smits, D.E.P. Muylaert, J.W. van Rijswijk, A. Vink, M.C. Verhaar, A. Driessen-Mol, L.A. van Herwerden, C.V.C. Bouten, J. Kluin, F.P.T. Baaijens, unpublished data). Therefore, the goal of the present study was to elucidate the initial response of circulating human immune cells to a MCP-1-loaded scaffold under physiological conditions of flow. We hypothesized that local delivery of exogenous MCP-1 would induce specific recruitment of reparatory CCR2^+^ cells from the circulatory system. Our results demonstrate that a local gradient of MCP-1 resulted in highly specific recruitment of CD14^+^ monocytes in static migration assays, whereas released MCP-1 had no effect on circulating hPBMC in terms of cell recruitment and adhesion in conditions of pulsatile flow.

The role of MCP-1 and its primary receptor CCR2 *in vivo* can be considered rather ambiguous. While MCP-1 is implicated in many cardiovascular pathological conditions, such as atherosclerosis and intima hyperplasia, it is indispensable for physiological tissue homoeostasis and angiogenesis [32–36]. It was shown that the MCP-1-CCR2 axis is involved in monocyte emigration from the bone marrow, recruitment to the site of inflammation, and migration into damaged tissue, but its mode of action remains incompletely understood 37. Therefore, we investigated the recruitment of monocytes towards MCP-1-loaded scaffolds in the current study, under static conditions as well as in flow. Considering the adverse effects of chronic overexpression of MCP-1, we opted for a short burst-release of MCP-1 from the scaffolds. We established the chemotactic potential of our hybrid MCP-1-loaded PCL/fibrin constructs after 4 hrs of incubation, which is in correspondence with the determined release curve, showing a burst-release of the bulk of the loaded MCP-1 within the first 3 hrs. A concentration of 20 ng/ml was sufficient to prompt specific migration. Capoccia *et al*. suggested that the angiogenic potential of monocytes is contained in the mon1 subset of circulating monocytes and they showed stimulation of angiogenesis in mice *via* CCR2-dependent signalling to the mon1 subset 38. Correspondingly, Cochain *et al*. suggested that an increase in the number of mon1 cells in circulation by MCP-1/CCR2 activation was the base of enhanced neovascularization in mice, while altered levels of mon3 had no effect on post-ischaemic neovascularization 39. Indeed, we did observe a predominant migration of mon1 towards our MCP-1-loaded scaffolds in terms of absolute cell numbers. However, interestingly, in terms of relative migration compared with the fibrin-seeded scaffolds, the most pronounced migration was represented by the mon2 subset, which has been reported to accumulate in injured tissue typically in the proliferative phase of healing, leading to improved outcome 40–42.

When subjected to a suspension of hPBMC in pulsatile flow, there was a marked increase in immediate monocyte recruitment in the MCP-1-loaded scaffold compared with the control scaffold, without evident selectivity in monocyte subsets. The increase in monocyte recruitment correlates strongly with the MCP-1 release rate as measured in conditions of pulsatile flow. This was accompanied by an up-regulation of CCR2 gene expression in the MCP-1-loaded scaffold group compared with the controls after 4 hrs. After the initial burst-release of MCP-1, however, the specific chemotactic effect of the scaffold was negated and cell adhesion to the scaffolds was similar in both groups. Overall, there was a gradual depletion of monocytes but not lymphocytes. Monocyte adhesion is known to be influenced by shear stress 12. Moreover, shear stress may have a differential effect on the specific monocyte subsets, as they exhibit distinctly different integrin presentation on their cell surface. Mon2 and mon3 mainly express integrin α_4_, while mon1 mainly express integrin β_2_. As integrin α_4_ is involved in the initial binding of monocytes to a substrate under influence of shear 43,44, this may explain the accelerated depletion of mon2 and mon3, compared with mon1, which we observed in flow cytometric analysis after 4 hrs of flow. Concurrently, gene expression analysis revealed a down-regulation of integrin β_2_, while integrin α_4_ expression remained relatively stable over time.

Recruited monocytes displayed monocyte-to-macrophage differentiation induced by the hybrid construct, regardless of MCP-1 loading. Macrophages are of vital importance for *in situ* cardiovascular tissue engineering and early macrophage presence was shown to determine late-term outcome in neotissue formation 45. Further classification of macrophage polarization state revealed a mixed population of predominantly CCR7^+^ macrophages (M1) with limited presence of CD163^+^ macrophages (M2), which was reflected in the gene expression analyses. This combination of inflammatory and reparative factors, will determine the local microenvironment for host cells colonizing the scaffold. This is in line with the recent findings by Willenborg *et al*., who described a mixed macrophage population, dominated by M1 macrophages, in the early-phase of tissue repair after skin wounding in mice 46.

PCL was used as the scaffold material based on its excellent biocompatibility and ease of processing. As such, PCL has numerous beneficial properties for large-scale use as vascular scaffold material and electrospun PCL grafts have been studied extensively in animal models 47–50. Apart from the material, the scaffold microstructure plays a profound role in cellular behaviour. The fibre diameter and pore size of electrospun scaffolds are interdependent and as such, fibre diameter and alignment influence the cell infiltration depth into the scaffold. A fibre diameter of ~10 μm as used in this study was shown to accommodate homogenous cell infiltration 22,51. The average fibre diameter and pore size have also been shown to influence (progenitor) cell orientation 52 and macrophage differentiation 53, respectively, as well as other processes that may impair or facilitate regeneration. Fibrin gel was employed here as a method to deliver the MCP-1 protein. Although we demonstrated that the fibrin itself does not have a chemotactic effect on the hPBMC subsets, *in vivo* the fibrin gel would probably act as a provisional matrix, offering important binding sites to circulating cells that adhere to and infiltrate into the scaffold.

The *in vitro* mesofluidics model used in the present study poses limitations in the sense that it does not include analogues for the bone marrow or splenic reservoirs. However, as the MCP-1-dependent extravasation of CCR2^+^ cells from the bone marrow has been well-established 16,54, we opted for an *in vitro* model without cell replenishment that allows us to follow the response of the initially recruited cells to the scaffold, rather than cell mobilization. The role of local MCP-1/CCR2 in monocyte arrest still raises some discrepancies 18,55. As compared with the landmark study by Roh *et al*., the released amount of MCP-1 per volume of blood/medium is lower in the current study (20 ng/ml *versus* 133 ng/ml). However, the absolute amount of MCP-1 (200 ng) is similar between studies, which implies that the local gradient of protein is comparable, at least initially. This is most relevant for local chemotaxis of the cells, which is the primary focus of the current study 21. Moreover, in a recent study by our group, we demonstrated enhanced *in situ* matrix formation and organization in rats using MCP-1-loaded vascular grafts with similar release kinetics as used in the current study (H. Talacua, A.I.P.M. Smits, D.E.P. Muylaert, J.W. van Rijswijk, A. Vink, M.C. Verhaar, A. Driessen-Mol, L.A. van Herwerden, C.V.C. Bouten, J. Kluin, F.P.T. Baaijens, unpublished data). Our results demonstrate that a local MCP-1 gradient resulted in recruitment of monocytes, whereas the protein no longer had an effect after release into the circulatory medium in our model. This is in line with previous findings, which showed that systemic MCP-1 is scavenged by circulating monocytes without affecting the functional responsiveness of the cells 56. Furthermore, the results of the current study do not show evidence for enhanced monocyte adhesion with the addition of exogenous MCP-1 in the scaffolds. This suggests that the presence of endogenously produced MCP-1 is required to stabilize anchorage *via* other adhesion proteins, as previously demonstrated 57, but that local increase of MCP-1 levels alone has no added value for monocyte arrest.

We focused here on the role of hPBMC only, rather than whole blood. *In vivo*, other circulating cell types, such as neutrophils and/or platelets, may play indirect or synergistic roles in the MCP-1-mediated response, which are currently not taken into account. However, as monocytes are considered the principle mediators of the regenerative response, this model allows for mechanistic studies with a high level of control. Furthermore, one of the main advantages of our methodology for translational studies is the use of human cells. This is of particular importance when studying the monocyte subsets, as these cells are known to be functionally different between species 4,5,7,59.

We conclude that in conditions of pulsatile flow, specific recruitment of circulating CCR2^+^ subsets is not the fundamental principle behind the previously observed improved neotissue formation in MCP-1-loaded constructs. Although our scaffold was proven to be highly chemotactic towards CD14^+^ monocytes, this selectivity was dependent on the release kinetics of MCP-1, as it was overruled by the effect of shear stress after the initial burst. This suggests that a controlled release of MCP-1 is essential in haemodynamic conditions and, therefore, should be considered in novel scaffold designs for *in situ* tissue engineering applications. These results emphasize the importance of testing in bio-mimicking conditions for future clinical translation.

## Supporting Information

**Figure S1**. Chemotaxis assays using MCP-1 directly dissolved in medium revealed enhanced migration of Mon1 and Mon2 subsets towards concentrations of 20 and 50 ng/ml. **Table S1**. Primer sequences encoding genes for qPCR analysis.
