# Supplementary material for: Shear flow affects selective monocyte recruitment into MCP-1-loaded scaffolds
Source: J Cell Mol Med. 2014 Aug 8;18(11):2176–88. doi: 10.1111/jcmm.12330 (PMC4224552; doi:10.1111/jcmm.12330)
Supplement: Supplementary file 1 — Figure S1. Chemotaxis assays using MCP-1 directly dissolved in medium revealed enhanced migration of Mon1 and Mon2 subsets towards concentrations of 20 and 50 ng/ml. Table S1. Primer sequences encoding genes for qPCR analysis. [file jcmm0018-2176-sd1.doc]

# Supplemental data to the manuscript entitled:

# Shear flow affects selective monocyte recruitment into MCP-1-loaded scaffolds

Anthal I.P.M. Smits^1,†^ , Virginia Ballotta^1,†^, Anita Driessen-Mol^1^, Carlijn V.C. Bouten^1,2^, Frank P.T. Baaijens^1,2,*^

^1^Eindhoven University of Technology

^2^Institute for Complex Molecular Systems (ICMS)

^†^ these authors contributed equally to this study

*Corresponding author: Frank P.T. Baaijens, P.O. Box 513,  5600 MB Eindhoven,  The Netherlands . Tel: +31 40 247 4888; fax +31 40 244 7355. E-mail: [f.p.t.baaijens@tue.nl](mailto:f.p.t.baaijens@tue.nl).

**Table S1. Primer sequences encoding genes for qPCR analysis.**

| **Primer** | **Symbol** | **Amplicon**  **size (bp)** | **Accession**  **number** | **Primer**  **Sequence (‘5-‘3)** |
| --- | --- | --- | --- | --- |
| **monocyte chemoattractant protein-1** | MCP-1 | 190 | NM_002982 | FW: CAGCCAGATGCAATCAATGCC  RV: TGGAATCCTGAACCCACTTCT |
| **tumor necrosis factor** | TNF | 91 | NM_000594 | FW: GAGGCCAAGCCCTGGTATG  RV: CGGGCCGATTGATCTCAGC |
| **interleukin 10** | IL10 | 112 | NM_000572 | FW: GACTTTAAGGGTTACCTGGGTTG  RV: TCACATGCGCCTTGATGTCTG |
| **chemokine (C-X-C motif) ligand 12** | CXCL12 | 48 | NM_000609 | FW: ATTCTCAACACTCCAAACTGTGC  RV: CTTCAGCCGGGCTACAATCTG |
| **chemokine (C-C motif) receptor 2** | CCR2 | 100 | NM_001123396 | FW: TGCAAAAAGCTGAAGTGCTTG  RV: CAGCAGAGTGAGCCCACAAT |
| **transforming growth factor, β1** | TGFB1 | 355 | NM_000660 | FW: GCAACAATTCCTGGCGATACCTC  RV: AGTTCTTCTCCGTGGAGCTGAAG |
| **integrin, β2** | ITGB2 | 187 | NM_000211 | FW: TGCGTCCTCTCTCAGGAGTG  RV: GGTCCATGATGTCGTCAGCC |
| **integrin, α4** | ITGA4 | 139 | NM_000885 | FW: CACAACACGCTGTTCGGCTA  RV: CGATCCTGCATCTGTAAATCGC |
| **chemokine (C-X3-C motif) receptor 1** | CX3CR1 | 226 | NM_001171174 | FW: TCACCGTCATCAGCATTGATAGG  RV: GTTTCCACATTGCGGAGCAC |
| **chemokine (C-X-C motif) receptor 4** | CXCR4 | 130 | NM_001008540 | FW: GCCTTATCCTGCCTGGTATTGTC  RV: GCGAAGAAAGCCAGGATGAGGAT |
| **vascular endothelial growth factor A** | VEGFA | 213 | NM_001025366 | FW: GCAGAATCATCACGAAGTGG  RV: GCATGGTGATGTTGGACTCC |
| **matrix metalloproteinase 9** | MMP9 | 224 | NM_004994 | FW: TGGGGGGCAACTCGGC  RV: GGAATGATCTAAGCCCAG |
| **interleukin 6** | IL6 | 45 | NM_000600 | FW: ACTCACCTCTTCAGAACGAATTG  RV: GTCGAGGATGTACCGAATTTGT |
| **interleukin 4** | IL4 | 102 | NM_000589 | FW: CAGTTCTACAGCCACCATGAG  RV: GTCGAGCCGTTTCAGGAATC |
| **interleukin 13** | IL13 | 121 | NM_002188 | FW: AGGCACACTTCTTCTTGGTCT  RV: GAGTCTCTGAACCCTTGGCT |
| **CD163 molecule** | CD163 | 137 | NM_004244 | FW: CACTATGAAGAAGCCAAAATTACCT  RV: AGAGAGAAGTCCGAATCACAGA |
| **mannose receptor, C type 1** | MRC1 | 114 | NM_002438 | FW: TGGGTTCCTCTCTGGTTTCC  RV: CAACATTTCTGAACAATCCTATCCA |
| **chemokine (C-C motif) receptor 7** | CCR7 | 106 | NM_001838 | FW: AAGCCTGGTTCCTCCCTATC  RV: ATGGTCTTGAGCCTCTTGAAATA |

The primer pairs for IL4, IL13, CD163, MRC1, and CCR7 were sequenced by PrimerDesign.


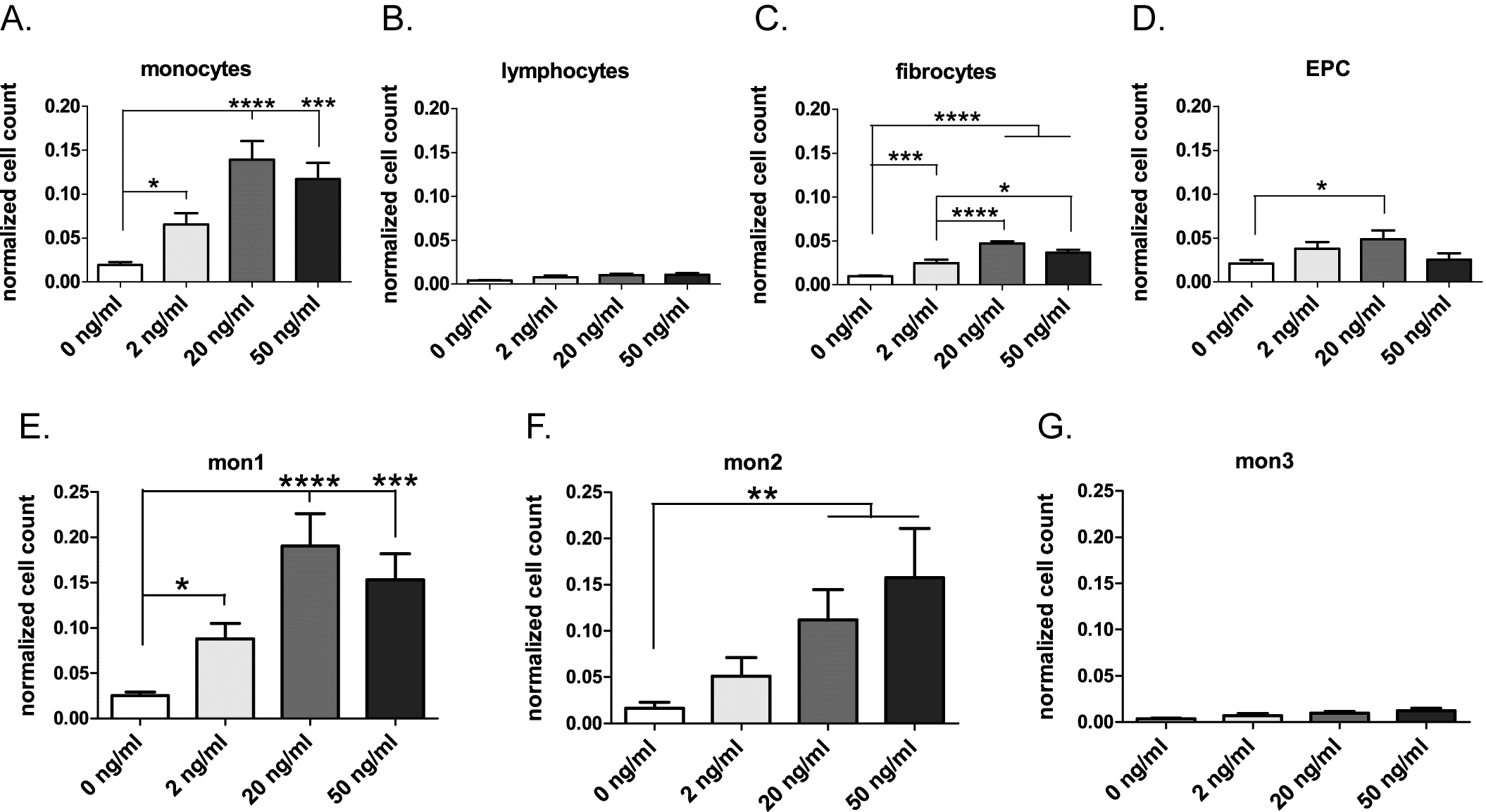


**Figure S1. Chemotaxis assays using MCP-1 directly dissolved in medium revealed enhanced migration of Mon1 and Mon2 subsets toward concentrations of 20 and 50 ng/ml.** (A-G) Quantification of migrated populations in response to various doses of MCP-1 dissolved in medium, without scaffolds. Cell counts were normalized per cell type on the initial cell count of that specific population. *P<0.05; **P<0.01; ***P<0.001; ****P<0.0001.
